# Correction: Jiang et al. Double-Gated Mamba Multi-Scale Adaptive Feature Learning Network for Unsupervised Single RGB Image Hyperspectral Image Reconstruction. *J. Imaging* 2026, *12*, 19

**DOI:** 10.3390/jimaging12020077

**Published:** 2026-02-11

**Authors:** Zhongmin Jiang, Zhen Wang, Wenju Wang, Jifan Zhu

**Affiliations:** College of Publishing, University of Shanghai for Science and Technology, Shanghai 200093, China; jzmn@usst.edu.cn (Z.J.);

## Text Correction

There were two errors in the original publication [1].

1. In the “Related Work” section, the second paragraph is missing the letter “I” and should be changed to “In”.

A correction has been made to 2. Related Work, Paragraph 2:

In supervised RGB-to-hyperspectral image reconstruction, the model is trained using a large dataset of paired RGB and hyperspectral images [20], where the RGB image is used to predict and reconstruct the hyperspectral image.

2. There is an error in the formula subscript in Formula (29).

A correction has been made to 3. Methodology, 3.3. Structure-Aware Smooth Loss Function, Formula (29):

(29) $$L_{curvature}=\frac{1}{N}\sum_{i,j}(\left|{\hat{I}}_{i+1,j}+{\hat{I}}_{i-1,j}+{\hat{I}}_{i,j+1}+{\hat{I}}_{i,j-1}-4{\hat{I}}_{i,j}\right|)$$

The authors state that the scientific conclusions are unaffected. This correction was approved by the Academic Editor. The original publication has also been updated.
